# Does screening and brief intervention for drug use in primary care increase receipt of substance use disorder treatment?

**DOI:** 10.1186/1940-0640-10-S2-O46

**Published:** 2015-09-24

**Authors:** Richard Saitz, Theresa Kim, Judith Bernstein, Debbie M Cheng, Jeffrey Samet, Christine Lloyd-Travaglini, Tibor Palfai, Jacqueline German

**Affiliations:** 1 Community Health Sciences and General Internal Medicine, Boston University Schools of Public Health & Medicine/Boston Medical Center, Boston, USA; 2 Community Health Sciences, Boston University Schools of Public Health & Medicine/Boston Medical Center, Boston, USA; 3 Biostatistics and General Internal Medicine, Boston University Schools of Public Health & Medicine/Boston Medical Center, Boston, USA; 4 General Internal Medicine and Community Health Sciences, Boston University Schools of Public Health & Medicine/Boston Medical Center, Boston, USA; 5 Data Coordinating Center, Boston University School of Public Health, Boston, USA; 6 Psychology and Brain Sciences, Boston University College of Arts and Sciences, Boston, USA

## Background

Little is known about the efficacy of "RT" (referral to treatment) for increasing receipt of substance use disorder (SUD) treatment by patients with unhealthy drug use identified by screening. We compared receipt of SUD treatment between baseline and 6 months across three randomized groups: no intervention and two different types of brief interventions.

## Material and methods

Adults presenting to a hospital-based primary care clinic with recent drug use (Alcohol, Smoking and Substance Involvement Screening Test [ASSIST] drug specific scores of ¿4) were enrolled in a randomized clinical trial comparing: (1) a 10-15 minute structured interview conducted by health educators (BNI), (2) a 30-45 minute intervention based on motivational interviewing by Masters-level counselors (MOTIV), or 3) no brief intervention. All received information on treatment resources. We assessed receipt of any SUD treatment in a statewide database. Logistic regression analyses adjusted for main drug (self-identified), drug dependence, and past SUD treatment.

## Results

Among 528 participants the main drug was marijuana (63%), cocaine (19%), and opioids (17%); 46% met 12-month drug dependence criteria (Composite International Diagnostic Interview Short Form); 18% had ASSIST scores (¿27) consistent with dependence (past 3-months). At 6 months, 14% (73/528) received any SUD treatment. There were no significant differences in SUD treatment receipt: BNI vs control (adusted odds ratio [AOR] 1.16, 95% Confidence Interval [CI] 0.59, 2.30, Hochberg adjusted p-value=0.66); MOTIV vs control (AOR 0.45, 95%CI: 0.21, 0.97, Hochberg adjusted p-value=0.08). There were no significant interactions between intervention and main drug, severity (ASSIST), or prior SUD treatment.

## Conclusions

Brief intervention did not increase receipt of SUD treatment in primary care patients. Future research should address how to make referral to treatment successful among screen-identified patients who could benefit from it.

